# Insights into the Injectivity and Propagation Behavior of Preformed Particle Gel (PPG) in a Low–Medium-Permeability Reservoir

**DOI:** 10.3390/gels10070475

**Published:** 2024-07-18

**Authors:** Hong He, Yuhang Tian, Lianfeng Zhang, Hongsheng Li, Yan Guo, Yu Liu, Yifei Liu

**Affiliations:** 1 College of Petroleum Engineering, Yangtze University, Wuhan 430100, China; hehong1103@163.com; 2 Key Laboratory of Drilling and Production Engineering for Oil and Gas, Wuhan 430100, China; 3 Research Institute of Exploration and Development, Sinopec Henan Oilfield Company, Nanyang 473132, China; 4 Henan Provincial Key Laboratory of Enhanced Oil Recovery, Nanyang 473132, China; 5 College of Petroleum Engineering, China University of Petroleum (East China), Qingdao 266555, China; liuyifei@upc.edu.cn

**Keywords:** preformed particle gel, injectivity and propagation behavior, matching relationship

## Abstract

Heterogeneous phase combined flooding (HPCF) has been a promising technology used for enhancing oil recovery in heterogeneous mature reservoirs. However, the injectivity and propagation behavior of preformed particle gel (PPG) in low–medium-permeability reservoir porous media is crucial for HPCF treatment in a low–medium-permeability reservoir. Thus, the injectivity and propagation behavior of preformed particle gel in a low–medium-permeability reservoir were systematically studied by conducting a series of sand pack flooding experiments. The matching factor (δ) was defined as the ratio of the average size of PPG particles to the mean size of pore throats and the pressure difference ratio (β) was proposed to characterize the injectivity and propagation ability of PPG. The results show that with the increase in particle size and the decrease in permeability, the resistance factor and residual resistance factor increase. With the increase in the matching factor, the resistance factor and residual resistance factor increase. The higher the resistance factor and residual resistance factor are, the worse the injectivity of particles is. By fitting the relationship curve, PPG injection and propagation standards were established: when the matching coefficient is less than 55 and β is less than 3.4, PPG can be injected; when the matching coefficient is 55–72 and β is 3.4–6.5, PPG injection is difficult; when the matching coefficient is greater than 72 and β is greater than 6.5, PPG cannot be injected Thus, the matching relationship between PPG particle size and reservoir permeability was obtained. This research will provide theoretical support for further EOR research and field application of heterogeneous phase combined flooding.

## 1. Introduction

With the continuous demand for oil and gas resources in China, increasing storage and production have been of importance [1,2,3]. As the mature oilfields have entered the stage of high water cut and high recovery degree, the reservoir heterogeneity becomes more serious and dispersed [4,5]. Conventional water flooding can hardly meet the demand for the stable production and increased production of crude oil [6,7,8,9,10]. Therefore, how to efficiently recover the remaining oil in porous media and greatly improve the oil recovery is a major problem that needs to be solved urgently. To solve the above problems, the preformed particle gel was developed by Shengli oilfield, which was compounded with polymer and surfactant to form heterogeneous phase combined flooding (HPCF) [11]. Realizing the transformation from the “liquid–liquid homogeneous phase” to the “liquid–solid heterogeneous phase”, which can solve the technical problems of serious reservoir heterogeneity, highly dispersed remaining oil, limited swept volume capacity of conventional continuous polymer and binary composite flooding system solutions, and being unable to greatly improve oil recovery. HPCF technology was firstly proposed by Shengli oilfield and the first pilot test was carried out in the Gudao Ng3 reservoir after polymer flooding. Before the implementation of HPCF technology, the water cut of the reservoir was 98.3%. After implementation, the oil production increased from 4.5 t/d to 81.2 t/d, and the water cut decreased from 98.3% to 79.8%, improving the recovery rate by 8.5%, with a final recovery rate of up to 63.6%. To date, the HPCF technology has been applied on a large scale in Sinopec, such as the Shengli oilfield and Henan oilfield, which will greatly improve oil recovery after polymer flooding. According to the statistics, after the industrial promotion and application of HPCF technology, significant oil increase effects have been achieved, with a cumulative oil production of 2.54 million tons and an expected increase in recovery rate of 12.5 percentage points, which can increase recoverable reserves by 17.4 million tons [12,13] and achieve certain effects of increasing oil production and decreasing water [14,15,16]. Preformed particle gel (PPG) is a micron-sized and flexible “soft solid” elastic particle with a unique molecular structure of “partial crosslinking and partial branching”, which is very important for oil displacement agents in HPCF [17,18,19,20,21]. However, the flow behavior of PPG in porous media was complex, which involves the migration, retention, plugging, and deformation mechanism of particles in porous media, and was also related to the interaction between particles and reservoir rocks and fluids [22,23,24,25,26,27].

The previous research mainly focused on the migration mechanism of PPG in porous media by using the micro-etching model and sand pack flow experiment, and demonstrated that the migration mode mainly depends on the change in pressure with time, the ratio of injected and produced particle diameters, and the residual resistance factor of each section of the core [28,29]. A transparent micro-model filled with transparent quartz sand was used to study the mechanism of pore throat scale migration, surface deposition release, and plugging deposition re-migration of microspheres in porous media. The decrease in salinity of displacement fluid and the increase in Darcy velocity were beneficial to the release of microspheres on the pore throat surface, and microspheres can plug the pore throat through three mechanisms: trapping plugging, stacking plugging and bridging plugging, and generating resistance to water flow [30]. At the same time, the retention of microsphere filters in porous media can enhance the plugging effect of microspheres. In addition, the migration mechanism of gel in porous media was studied by core laboratory experiments and establishing the functional relationship between the resistance factor, residual resistance factor, and different influencing factors [31]. The main variables of PPG design were particle size/permeability ratio and vertical/horizontal permeability ratio. The effects of concentration, velocity, and particle size of the gel particle suspension on the pore plugging process were studied through core flow experiments. The results show that the conditions leading to pore throat plugging in a high-permeability layer can be quantified by related dimensionless groups, such as the Reynolds number and the ratio of pore throat diameter to particle size. When the Reynolds number and the ratio of pore throat to particle size are below the power law, plugging will occur [32].

In addition, aiming for a low–medium-permeability reservoir, there was little research on the matching relationship of PPG, the matching chart formed was not perfect, and the seepage law of PPG under medium- and low-permeability conditions was not well understood [33,34]. How to choose PPG type matching with the rock pore throat and establish appropriate seepage resistance expansion sweep volume was still unclear. Therefore, because of the characteristics of the II4-5 reservoir in Shuanghe North Block, Henan Province, such as the strong heterogeneity, large permeability variation range, many development layers, and large pore throat range, this study used dry powder particle size to form a chart, which was beneficial to the application and selection of particle size in the field. Based on the sand pack model, the core seepage experiment was carried out, and the seepage law of different dry powder particle sizes under the conditions of medium and low permeability were analyzed, to obtain a chart suitable for PPG matching with low–medium-permeability reservoirs [35,36,37].

## 2. Results and Discussion

### 2.1. The Particle Size Distribution of PPG Determination

**Figure 1:**
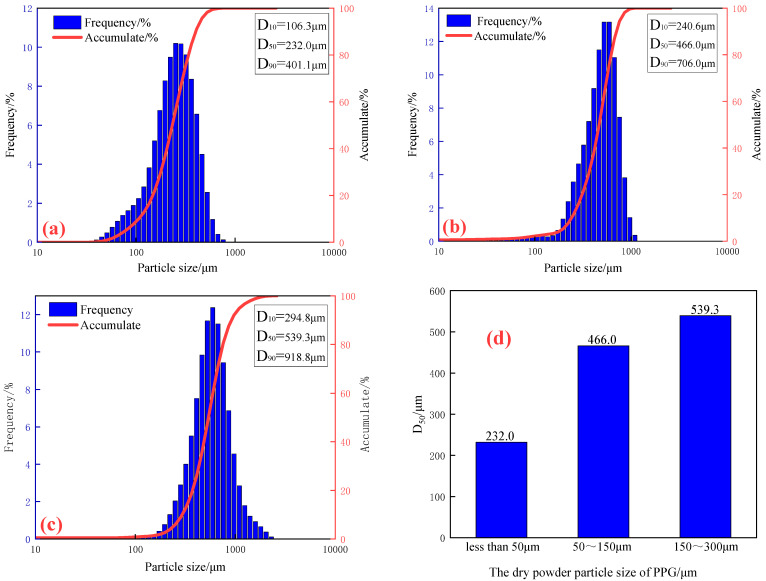
Particle size and distribution of performed particle gel after swelling. (**a**) Dry powder was less than 50 μm; (**b**) dry powder was 50~150 μm; (**c**) dry powder was 150~300 μm; (**d**) median particle size.

Three screened preformed particle gels with different particle sizes were prepared into a suspension system of 1000 mg/L, and the particle size and distribution of PPG were measured by a Bettersize2600 laser particle size analyzer, as shown in Figure 1.

As is shown in Figure 1, with the increase in the particle size of PPG dry powder, the particle size after swelling gradually increases. The median particle size of PPG dry powder below 50 μm was 232 μm, that of PPG dry powder between approximately 50 μm and 150 μm was 466 μm, and that of PPG dry powder between approximately 150 μm and 300 μm was 539.3 μm. The swelling multiple was three to four times.

### 2.2. The Seepage Characteristics of PPG in Porous Media

The above-mentioned gel particle dry powders with different particle sizes were prepared into a dispersion system of 1000 mg/L, and injected at a fixed speed of 0.5 mL/min by using sand pack models with different permeabilities. The flow behavior in porous media was studied, the pressure changes at different stages were detected, and the resistance factor and residual resistance factor were calculated.

#### 2.2.1. Pressure Change Characteristics

**Figure 2:**
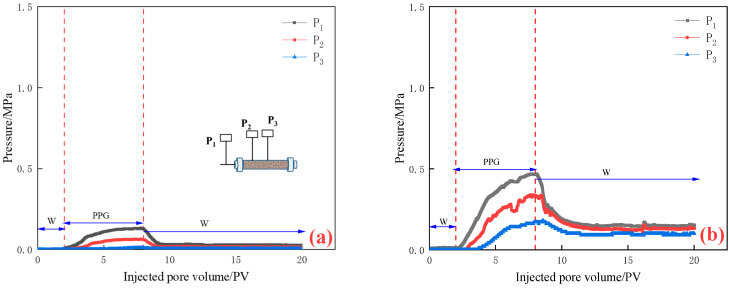

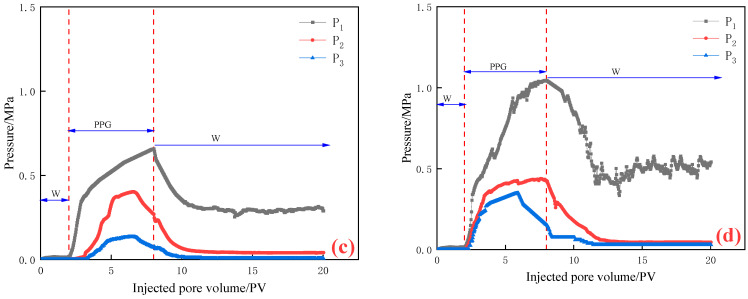
Pressure curve of PPG with different permeabilities less than 50 μm: (**a**) K = 1090 mD; (**b**) K = 505 mD; (**c**) K = 310 mD; and (**d**) K = 195 mD.

(1) The particle size of PPG dry powder was less than 50 μm. Seepage pressure changes of different permeabilities are shown in Figure 2.

As can be seen from Figure 2, in the initial water flooding stage (0–2 PV), the injection end pressure P_1_, the pressure P_2_ at pressure measuring point 1, and the pressure P_3_ at pressure measuring point 2 all remained at low values, and the injection end pressure P_1_ showed an upward trend when PPG was injected. This is because the injected particles of PPG kept accumulating at the end face, which led to the pressure rising. With the decrease in permeability, the steeper the pressure curve at the injection end, the greater the rising amplitude. The pressures P_2_ and P_3_ at the pressure measuring point were different with the change in permeability. When the permeability was greater than 500 mD, the pressure increased continuously with the increase in injection quantity, and when the permeability was less than 300 mD, it first increased and then decreased with the increase in injection quantity. With the decrease in permeability, the injection end pressure P_1_ and pressure measuring point pressures P_2_ and P_3_ increased continuously. In the subsequent water flooding stage, the pressure P_1_ at the injection end showed a trend of “first decreasing and then fluctuating”, which indicated that due to the later stage of water flooding, there was no subsequent injection of PPG particles, and the accumulated PPG began to be washed away and moved to the deep part, resulting in a significant pressure drop. Later, when PPG encountered small pores and was squeezed, it was deformed and blocked in the pores. With the increase in pressure, PPG was deformed and broken, and the subsequent PPG particles kept falling. The pressures P_2_ and P_3_ at pressure measuring points showed a trend of “decreasing first and then stabilizing”. With the increase in permeability, the injection pressure at the pressure measuring point decreased.

**Figure 3:**
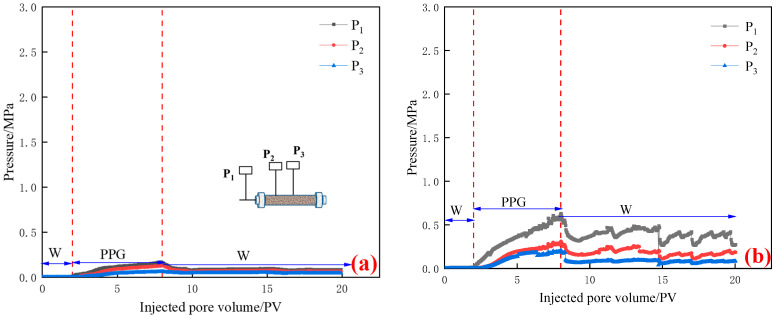

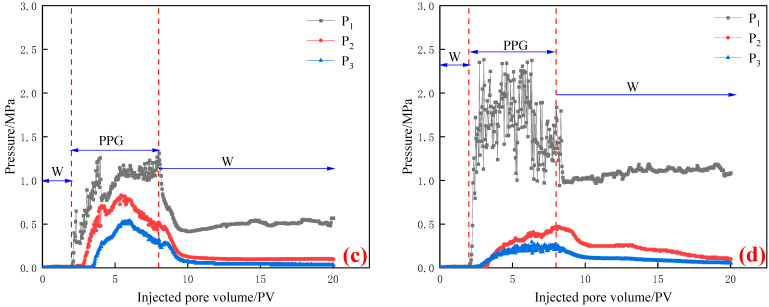
Pressure curve of PPG with different permeabilities of 50~150 μm: (**a**) K = 980 mD; (**b**) K = 490 mD; (**c**) K = 295 mD; and (**d**) K = 210 mD.

(2) The particle size of PPG dry powder was 50~150 μm. Seepage pressure changes of different permeabilities are shown in Figure 3.

As is shown in Figure 3, in the initial water flooding stage (0–2 PV), the injection end pressure P_1_, and the pressure measuring point pressures P_1_ and P_2_ demonstrated little change. In the PPG injection stage, the injection end pressure increased with the injection of particles. When the permeability was lower than 300 mD, the fluctuation of “up–down–up” began to rise during the injection process. This was because the particles were piled up at the end face, resulting in no blockage, and the pressure began to decrease with the subsequent PPG. When the permeability of pressure measuring points P_2_ and P_3_ was greater than 500 mD, they were always rising with the injection of PPG; when the permeability was less than 300 mD, they showed a trend of “rising first and then falling” with the injection of PPG. With the decrease in permeability, the pressure P_1_ at the injection end gradually increased, but the pressures P_2_ and P_3_ at the pressure measuring points first increased and then decreased. In the subsequent water flooding stage, the injection end pressure P_1_ and the pressure at the pressure measuring points P_2_ and P_3_ also showed a trend of “first decreasing and then fluctuating”.

**Figure 4:**
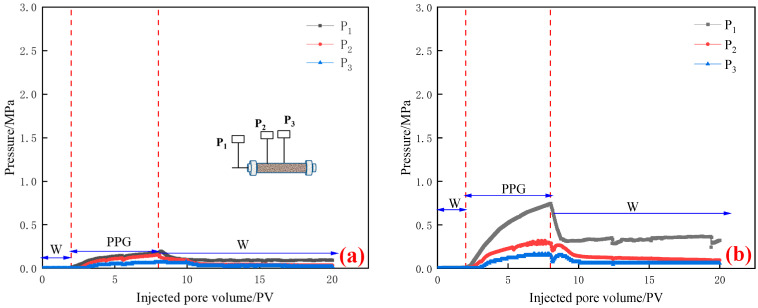

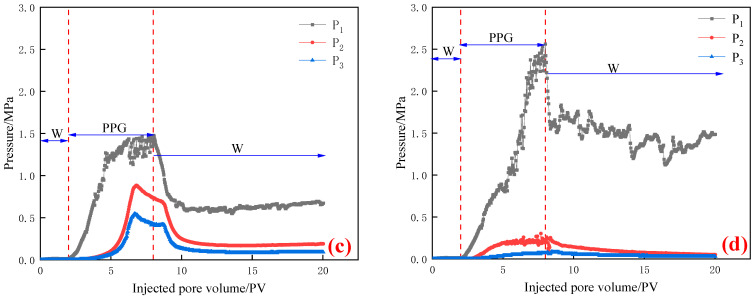
Pressure curve of PPG with different permeabilities of 150~300 μm: (**a**) K = 1020 mD; (**b**) K = 495 mD; (**c**) K = 310 mD; and (**d**) K = 190 mD.

(3) The particle size of PPG dry powder was 150~300 μm. Seepage pressure changes of different permeabilities are shown in Figure 4.

As is shown in Figure 4, in the initial water flooding stage (0–2 PV), the three pressures were not pressurized, and the pressure had no obvious change. In the injection stage of PPG, with the continuous injection of PPG, the pressure at the injection end kept rising. When the permeability was less than 500 mD, the injection stage was easily blocked due to the large particle size, so it easily rose in a “rise–fall–rise” fluctuation manner. When the permeability was 1000 mD, the pressure points P_2_ and P_3_ increased continuously with the increase in injection quantity; when the permeability was lower than 500 mD, the pressure points P_2_ and P_3_ first increased and then decreased with the increase in injection quantity. Like PPG with a particle size of 50–150 μm, with the decrease in permeability, the pressure at the injection end P_1_ gradually increased, but the pressure at the pressure measuring points P_2_ and P_3_ first increased and then decreased. In the subsequent water flooding stage, the injection end pressure P_1_ and the pressure at the pressure measuring points P_2_ and P_3_ also showed a trend of “first decreasing and then fluctuating”.

#### 2.2.2. Analysis of Resistance Factor and Residual Resistance Factor

The resistance factor and residual resistance factor of PPG in core seepage were calculated according to the pressure difference in injection end at different stages, and its plugging property and injection performance were evaluated. The flow parameters are shown in Table 1.

**Figure 5:**
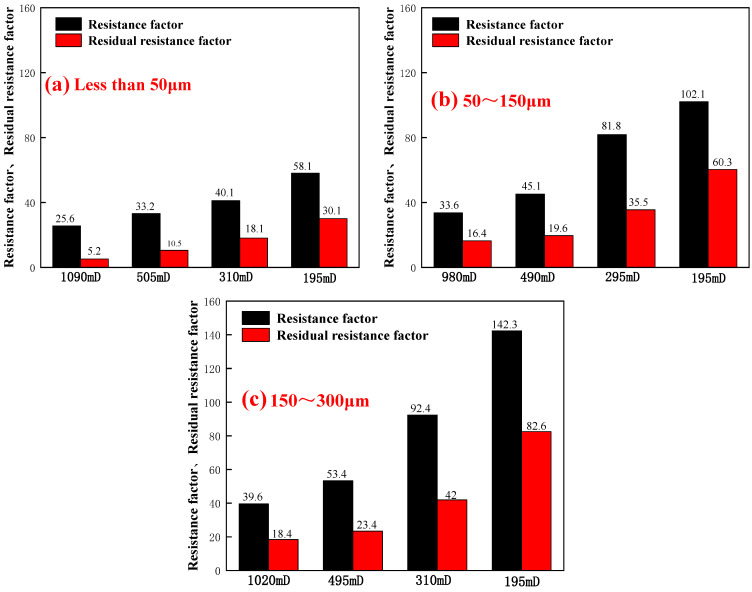
Resistance factor and residual resistance factor under different particle sizes and permeabilities: (**a**) less than 50 μm; (**b**) 50~150 μm; and (**c**) 150~300 μm.

**Figure 6:**
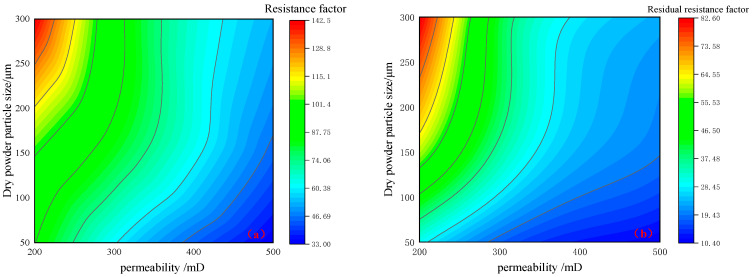
Three-dimensional field diagram: (**a**) resistance factor; (**b**) residual resistance factor.

As is shown in Table 1 and Figure 5, for preformed particle gel with the same particle size, with the decrease in permeability, the greater the PPG flooding pressure, the greater the subsequent water flooding pressure, and the higher the resistance factor and residual resistance factor. Because, the lower the permeability, the smaller the average pore throat radius of the core, and the more difficult it was for PPG to be injected into the deep part of the core and accumulated at the end face, resulting in pressure hold-up. At the same time, the smaller the pore, the easier it was for PPG to be adsorbed at the pore, resulting in increased seepage resistance, which makes it difficult to reduce the subsequent water flooding pressure and increase the residual resistance factor. Compared with Figure 5a–c, under the same permeability, with the increase in particle size, the injection pressure and subsequent water flooding pressure during PPG flooding also increased, and the resistance factor and residual resistance factor also increased. This was because the larger the particle size, the more difficult it was to pass through the pores, the easier it was to block at the end face, and the more difficult it was to deform and migrate to the deep through the pores. Furthermore, as is shown in Figure 6, it can be observed from the field plot that with the increase in particle size and permeability, the resistance factor and residual resistance factor increased. Therefore, the lower the permeability, the worse the injectability of preformed particle gel and the stronger the plugging ability. The smaller the particle size, the better the injectability and the weaker the plugging property of PPG.

### 2.3. The Matching Relationship between PPG Particle Size and Reservoir Permeability

The injectivity of PPG was related to particle size and core permeability (that is, the matching relationship between PPG and pore throat). To establish the evaluation index of gel particle injectability, the transport ability of PPG in the pore throat was summarized and the matching relationship was analyzed, which provides theoretical support for gel particle size and reservoir permeability in the Henan Oilfield.

#### 2.3.1. Analysis of the Law of Injectivity and Propagation

**Figure 7:**
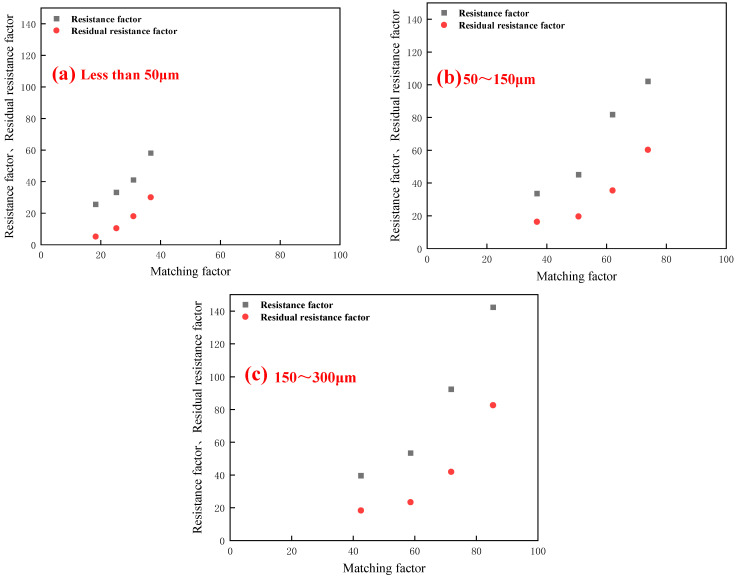
Resistance factor and residual resistance factor under different matching factors: (**a**) less than 50 μm; (**b**) 50~150 μm; and (**c**) 150~300 μm.

To further clarify the influence of core permeability and particle size on the injection performance of PPG, the matching factor was introduced to characterize the matching law between PPG and the core, as shown in Table 2, and the matching law was better defined by establishing the relationship diagram between the matching factor and the resistance factor and the residual resistance factor, as shown in Figure 7.

As is shown in Table 2, with the decrease in permeability, the matching factor increases, and the particle size of preformed particle gel increases, so does the matching factor. As is shown in Figure 7, with the increase in the matching factor, the resistance factor and residual resistance factor of preformed particle gel increase.

**Figure 8:**
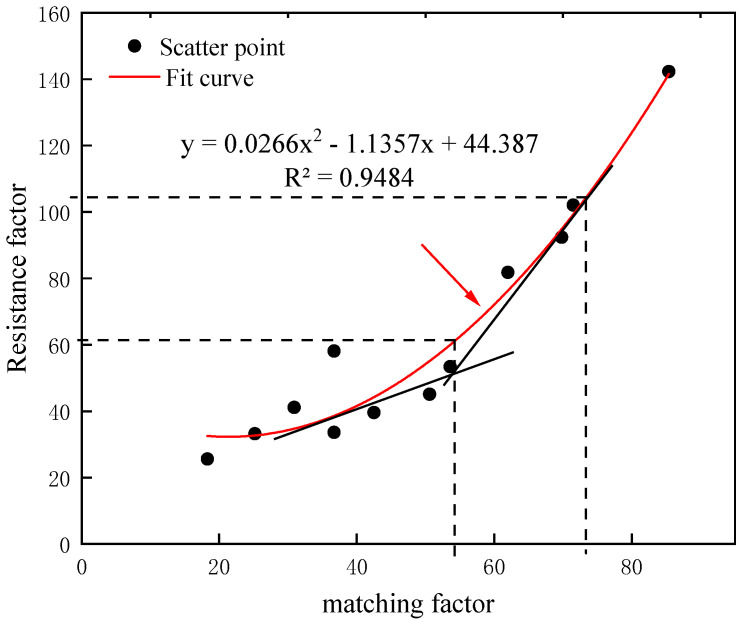
The curve of the fitting relationship between matching factor and resistance factor.

To quantify the relationship between the matching factor and the injectivity of preformed particle gel, the curve fitting between the matching factor and the resistance factor is shown in Figure 8. As is shown in the figure, the intersection of the two tangents corresponds to a matching factor of 55 and a corresponding resistance factor of 62. When the matching factor was less than 55, the curve rose gently, but the resistance factor did not increase much, so that particles could be injected into the sand pack smoothly. When the matching factor was greater than 72, the corresponding resistance factor was 103. At this time, the curve rose rapidly, and the particles were blocked at the end face of the core, making it difficult to migrate to the deep. Therefore, it was defined that when the matching factor was less than 55, particles could be injected smoothly; when the matching factor was 55~72, it was difficult to inject particles; and when the matching factor was greater than 72, particles could not be injected.

To demonstrate the propagation of PPG by analyzing the pressure difference ψ_P_ and the pressure difference ratio β, its parameters are shown in Table 3.

As can be seen from the table, for PPG with the same particle size, with the decrease in permeability, the differential pressure ψ_P1_, ψ_P2_, and differential pressure ratio β increase, indicating that the lower the permeability, the more easily the particles were blocked at the end face and the front end of the sand pack, and the worse the propagation, resulting in P_1_ > P_2_ > P_3_. The lower the permeability, the greater the differential pressure and differential pressure ratio, the more serious the particle end-face-blocking phenomenon, and the greater the pressure at the injection end.

**Figure 9:**
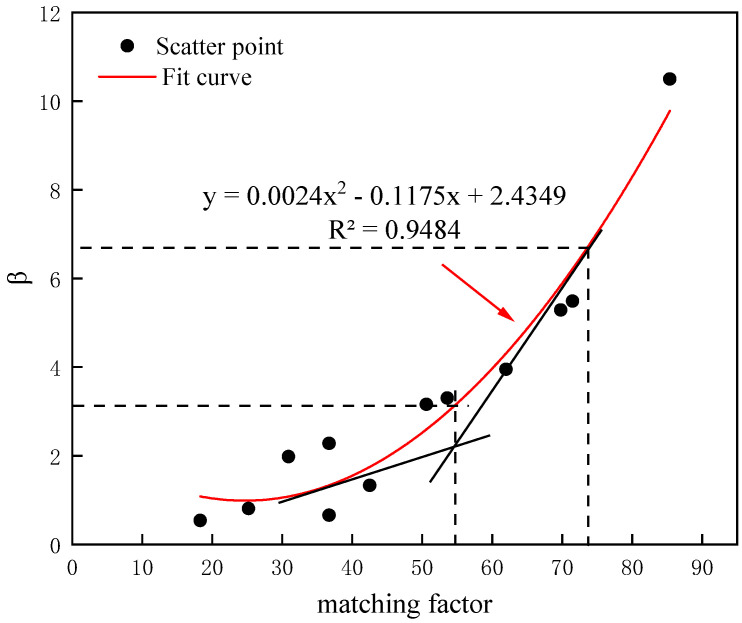
The curve of the fitting relationship between the matching factor and β.

To specifically quantify the relationship between the β value and gel particle propagation, the curve of the matching factor and β value was fitted, as shown in Figure 9. As is shown in the figure, the β value corresponding to the intersection of two tangents was 3. When β was less than 3, the curve rose gently, and when β was greater than 6, the curve rose rapidly. Therefore, it was defined that when β was 0~3.4, the propagation was excellent; when β was 3.4~6.5, the propagation was medium; and when β was greater than 6.5, the propagation was poor.

#### 2.3.2. Evaluation Criteria of PPG Injectivity and Propagation Were Established 

The injectability standards of PPG with different particle sizes at different permeabilities were determined through the characteristics of pressure change, resistance factor, and residual resistance factor, as well as β value and matching factor. Divided into the following three categories:

(1) Injectable: After water flooding, PPG flooding was carried out, and the pressure rose at first and then became flat. After water flooding, it was easy to move in the hole, with small values of resistance factor and residual power factor, matching factor δ less than 55, and β less than 3.4, with excellent propagation. In addition, particles can pass through the hole smoothly or deform, so that they can be injected and move at the same time.
(2) Difficulty in injection: after water flooding: PPG flooding was carried out, and the pressure rose rapidly. After accumulation to a certain extent, it becomes stable after being broken. After subsequent water flooding, it deforms or breaks through the pore, and then the process of plugging–crushing–plugging is repeated. The matching factor δ was 55~72, β was 3.4~6.5, and the propagation was moderate.
(3) Non-injectability: After water flooding, PPG flooding made it difficult to inject particles, and the pressure rose rapidly. After breaking through the deformation, the pressure dropped slightly, the resistance factor and residual resistance factor were large, the matching factor was greater than 72, β was greater than 6, and the propagation was poor. Furthermore, the particles were blocked at the end face, making it difficult to migrate to the deep.

#### 2.3.3. The Matching Relationship between PPG Particle Size and Reservoir Permeability

**Figure 10:**
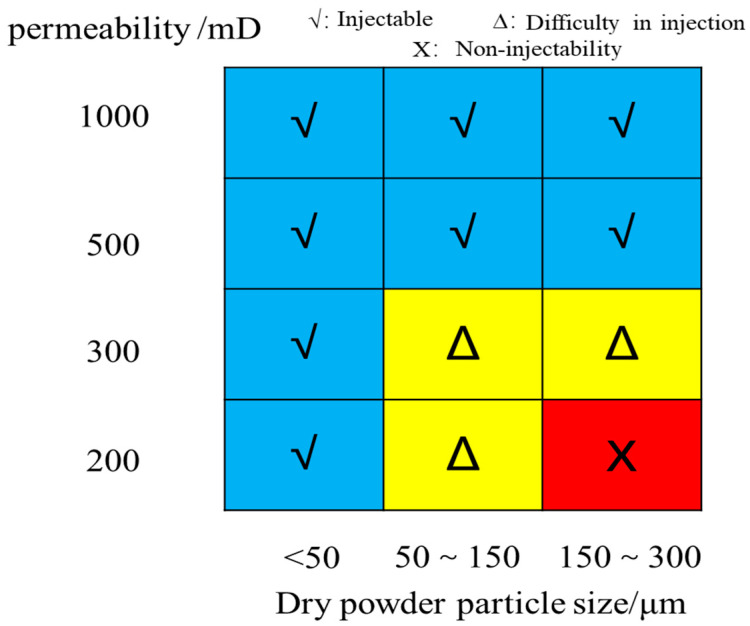
Matching chart of particle size and permeability of PPG dry powder.

From the above analysis, it can be concluded that the matching chart between PPG particle size and permeability can be obtained based on the characteristics of pressure changes, resistance factor and residual resistance factor, β value, and matching factor, as well as the changes in PPG injection standards, as shown in Figure 10.

Therefore, for PPG with dry powder of less than 50 μm, it can be injected smoothly at the permeability of 200–1000 mD; for PPG with dry powder of 50~150 μm, it becomes difficult to inject at the permeability of less than 300 mD; for PPG with dry powder of 150~300 μm, it becomes difficult to inject at the permeability of less than 300 mD; and it cannot be injected at the permeability of less than 200 mD.

## 3. Conclusions

In this study, the injectivity and propagation of PPG in low–medium-permeability reservoirs were studied. The main findings are as follows:

(1) For PPG gel particles, with the increase in particle size and the decrease in permeability, the greater the resistance factor and residual resistance factor, and the worse the injectability of particles. With the increase in the matching factor, the resistance factor and residual resistance factor increase.
(2) The evaluation criteria for PPG injectability and propagation were divided. When the matching coefficient is less than 55 and β is less than 3.4, PPG can be injected; when the matching coefficient is 55–72 and β is 3.4–6.5, PPG injection is difficult; when the matching coefficient is greater than 72 and β is greater than 6.5, PPG cannot be injected.
(3) Thus, a matching chart of PPG permeability and particle size was obtained, providing injection standards for on-site PPG applications.

## 4. Materials and Methods

### 4.1. Materials

The different particle sizes of dry preformed particle gel used in this study was provided by the Henan Oilfield Exploration Research Institute. The particle size of dry powder was less than 50 μm, 50–150 μm, and 150–300 μm. The formation brine salinity of 5002 mg·L^−1^ and its ion composition are shown in Table 4.

### 4.2. Methods

#### 4.2.1. Determination of the Particle Size of the Gel Particle Suspension

The morphological characteristics, particle size, and distribution of swollen PPG particles were characterized by the Bettersize2600 laser particle size analyzer, which provided basic parameters for further study on the flow behavior of PPG in porous media.

#### 4.2.2. The Seepage Behavior of PPG Particles in Porous Media

**Figure 11:**
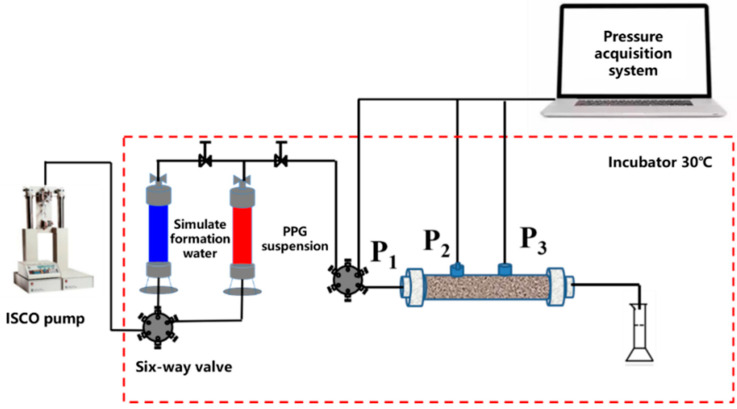
Flow chart of core displacement experimental device.

The experiment was carried out at 70 °C and normal pressure, and the flow rate of the constant pressure pump was 0.5 mL·min^−1^. The experimental process is shown in Figure 11, and the experimental steps were as follows: (1) The sand pack (Φ2.5 cm × 30 cm) was packed by using quartz sand with different mesh and its pore volume and porosity were measured. (2) The permeability of the sand pack core was measured by using simulated formation brine at a flow rate of 1.0 mL·min^−1^. (3) Water flooding was conducted until the injection pressure reached stable at 2.0 PV, then 6.0 PV of PPG suspension was injected and subsequent water flooding was conducted to 20 PV. The change in pressure was recorded versus the injected pore volume by using a pressure acquisition system. (4) The resistance factor (Fr) and residual resistance factor (Frr) of the displacement fluid were calculated according to the pressure change at the injection end [38,39]. The calculation formulas of resistance factor and residual resistance factor were as follows:

(1) $$Fr=\frac{\Delta P_{\mathrm{PPG}}}{\Delta P_{\mathrm{wb}}}$$

(2) $$F\mathrm{rr}=\frac{\Delta P_{\mathrm{wa}}}{\Delta P_{\mathrm{wb}}}$$

where ΔP_PPG_ was the pressure difference after injecting PPG particles, MPa; ΔP_wa_ and ΔP_wb_ were the subsequent water flooding pressure difference after injecting PPG particles and the initial water flooding pressure difference before injecting PPG particles, MPa.

#### 4.2.3. PPG Injectivity and Propagation Performance Evaluation

The injectivity and propagation of PPG were further analyzed by defining the value of matching factor δ and propagation capacity β, and the gel particle matching chart suitable for the II4-5 reservoir in Shuanghe North Block of Henan Province was obtained.

(1) The matching factor δ was introduced as the ratio of the median particle size of PPG after swelling to the average pore throat diameter to characterize the matching law between PPG and the core [40]. The average pore throat diameter and matching factor can be calculated by the formula as follows:

(3) $$K=\frac{{\emptyset r}^{2}}{8\tau^{2}}$$

(4) $$\delta =\frac{2r}{D_{50}}$$

where K was the core permeability, μm^2^; Φ was porosity, a dimensionless quantity; r was the average pore throat radius, μm; τ was the tortuosity, dimensionless quantity, taking 1.2 D_50_ as the median particle size of PPG after swelling, μm; and δ was the matching factor, dimensionless quantity.

(2) By analyzing the pressure difference and pressure difference ratio of each pressure measuring point, the transport and migration ability of PPG in the core was evaluated.

ψ_P1_ = P_1_ − P_2_ (5)

ψ_P2_ = P_2_ − P_3_ (6)

(7) $$\beta =\frac{\psi_{P1}}{\psi_{P2}}$$

where P_1_, P_2_, and P_3_ were the pressure values at each pressure measurement point, MPa; ψ_P1_, ψ_P2_ were the pressure differences between successive measuring points, MPa; and β was the pressure difference ratio, dimensionless quantity.

## Figures and Tables

**Table 1:** Flow parameters of PPG with different particle sizes and different permeabilities.

Number	PPG Dry Powder Particle Size	Permeability/mD	Water Flooding Pressure/MPa	PPG Flooding Pressure/MPa	Subsequent Water Flooding Pressure/MPa	Resistance Factor	Residual Resistance Factor
1	Less than 50 μm	1090	0.0045	0.128	0.026	25.6	5.2
2		505	0.014	0.465	0.147	33.2	10.5
3		310	0.016	0.658	0.269	41.1	18.1
4		195	0.018	1.046	0.425	58.1	30.1
5	50~150 μm	980	0.005	0.168	0.082	33.6	16.4
6		490	0.014	0.631	0.274	45.1	19.6
7		295	0.016	1.31	0.568	81.8	35.5
8		210	0.018	1.838	1.085	102.1	60.3
9	150~300 μm	1020	0.005	0.198	0.092	39.6	18.4
10		495	0.014	0.748	0.327	53.4	23.4
11		310	0.016	1.478	0.672	92.4	42
12		190	0.018	2.561	1.486	142.3	82.6

**Table 2:** Parameters of sand pack cores.

Number	PPG Dry Powder Particle Size	Permeability/mD	Average Pore Throat Diameter/μm	Matching Factor δ
1	Less than 50 μm	1090	12.953	18.3
2		505	9.214	25.2
3		310	7.555	30.9
4		195	6.304	36.7
5	50~150 μm	980	12.703	36.7
6		490	9.204	50.6
7		295	7.515	62.0
8		210	6.314	71.5
9	150~300 μm	1020	12.903	42.5
10		495	9.104	53.6
11		310	7.615	69.8
12		190	6.114	85.4

**Table 3:** Pressure difference and pressure difference ratio at pressure measuring point of the sand pack.

Number	PPG Dry Powder Particle Size	Permeability/mD	P_1_/MPa	P_2_/MPa	P_3_/MPa	ψ_P1_/MPa	ψ_P2_/MPa	β
1	Less than 50 μm	1090	0.128	0.09	0.02	0.038	0.07	0.54
2		505	0.465	0.333	0.17	0.132	0.163	0.81
3		310	0.658	0.269	0.072	0.389	0.197	1.97
4		195	1.046	0.425	0.153	0.621	0.272	2.28
5	50~150 μm	980	0.168	0.126	0.062	0.042	0.064	0.66
6		490	0.631	0.309	0.207	0.322	0.102	3.12
7		295	1.31	0.52	0.32	0.79	0.2	3.95
8		210	1.838	0.46	0.209	1.378	0.251	5.49
9	150~300 μm	1020	0.198	0.125	0.07	0.073	0.055	1.33
10		495	0.748	0.289	0.15	0.459	0.139	3.3
11		310	1.478	0.336	0.12	1.142	0.216	5.29
12		190	2.561	0.25	0.03	2.311	0.22	10.50

**Table 4:** Composition of simulated formation brine in the Henan oilfield.

Ion Type	Na^+^, K^+^	Ca^2+^	Mg^2+^	Cl^−^	SO_4_^2−^	HCO^3−^	TDS
Ion composition (mg·L^−1^)	1601	14	7.5	1172	391.5	1816	5002

## Data Availability

All data and materials are available on request from the corresponding author. The data are not publicly available due to ongoing researches using a part of the data.
